# The Influence of Dynamic Taping on Landing Biomechanics after Fatigue in Young Football Athletes: A Randomized, Sham-Controlled Crossover Trial

**DOI:** 10.3390/bioengineering11060631

**Published:** 2024-06-20

**Authors:** Chih-Kuan Wu, Yin-Chou Lin, Ya-Lin Chen, Yi-Ping Chao, Tsung-Hsun Hsieh

**Affiliations:** 1 Department of Physical Medicine and Rehabilitation, Chang Gung Memorial Hospital, Linkou, Taoyuan 33305, Taiwan; a9227@adm.cgmh.org.tw (C.-K.W.); sirius@adm.cgmh.org.tw (Y.-C.L.); 2 Center of Comprehensive Sports Medicine, Chang Gung Memorial Hospital, Linkou, Taoyuan 33305, Taiwan; 3 School of Physical Therapy and Graduate Institute of Rehabilitation Science, Chang Gung University, Taoyuan 33302, Taiwan; 4 Department of Health Management and Enhancement, Open University of Kaohsiung, Kaohsiung 81249, Taiwan; 5 Department of Athletic Training & Health, National Taiwan Sport University, Taoyuan 33301, Taiwan; eileen@ntsu.edu.tw; 6 Department of Computer Science and Information Engineering, Chang Gung University, Taoyuan 33302, Taiwan; yiping@mail.cgu.edu.tw; 7 Neuroscience Research Center, Chang Gung Memorial Hospital, Linkou, Taoyuan 33305, Taiwan; 8 Healthy Aging Research Center, Chang Gung University, Taoyuan 33305, Taiwan

**Keywords:** dynamic taping, landing biomechanics, fatigue, athletes

## Abstract

Fatigue is believed to increase the risk of anterior cruciate ligament (ACL) injury by directly promoting high-risk biomechanics in the lower limbs. Studies have shown that dynamic taping can help normalize inadequate biomechanics during landings. This study aims to examine the effects of dynamic taping on landing biomechanics in fatigued football athletes. Twenty-seven high-school football athletes were recruited and randomly allocated to groups of either active taping or sham taping, with a crossover allocation two weeks later. In each group, the participants underwent a functional agility short-term fatigue protocol and were evaluated using the landing error scoring system before and after the fatigue protocol. The landing error scoring system (LESS) scores in the sham taping group increased from 4.24 ± 1.83 to 5.36 ± 2.00 (*t* = −2.07, *p* = 0.04, effect size = 0.61). In contrast, the pre–post difference did not reach statistical significance in the active taping group (from 4.24 ± 1.69 to 4.52 ± 1.69, *t* = −1.50, *p* = 0.15, effect size 0.46). Furthermore, the pre–post changes between the sham and active taping groups were statistically significant (sham taping: 1.12 ± 1.20; active taping: 0.28 ± 0.94, *p* = 0.007). Dynamic taping, particularly using the spiral technique, appeared to mitigate faulty landing biomechanics in the fatigued athletes by reducing hip and knee flexion and increasing hip internal rotation during landing. These results suggest that dynamic taping can potentially offer protective benefits in landing mechanics, which could further be applied to prevent ACL injuries in fatigued athletes.

## 1. Introduction

An anterior cruciate ligament (ACL) injury presents a significant setback for competitive athletes. Studies indicate that even after surgery and extensive rehabilitation, only 55% of athletes return to competitive sports [1], with two-thirds playing at their pre-injury level post-ACL reconstruction surgery [2]. The ensuing functional limitations, financial burdens, and rehabilitation programs compound the stress for injured athletes. Non-contact ACL injuries often occur during movements involving cutting, deceleration, and landing, with the highest incidence rates seen in basketball, handball, and football [3,4,5]. Moreover, research has identified variations in ACL injuries across genders, ages, and ethnicities; for instance, a study in Asia found peak injury rates among high-school male and female athletes in the 11th grade [3]. 

ACL injuries are influenced by both extrinsic and intrinsic factors. Extrinsic factors, which are modifiable, encompass the type of sport, level of competition, playing environment, and equipment [6]. Intrinsic factors can be further categorized as non-modifiable (e.g., genetics, anatomical characteristics, female gender) or modifiable (e.g., biomechanical factors like muscle strengthening, jumping, and landing techniques) [7,8,9,10,11,12,13]. Various biomechanical risk factors have been identified, including increased knee and hip joint internal rotation, hip adduction, anterior tibial shear force, decreased knee flexion during landing, and increased knee valgus [14,15,16,17,18,19].

Fatigue has been found to alter lower-limb biomechanics, decreasing hip and knee flexion, increasing anterior tibial shear force, and altering the knee valgus and internal rotation angles, thereby increasing the load on the ACL [20,21,22]. To investigate fatigue’s effect on lower-limb biomechanics, researchers have devised several protocols, such as the functional agility short-term fatigue protocol, which employs football-specific drills to induce fatigue. This protocol has shown alterations in landing biomechanics during stop jumping tests, including decreased hip and knee flexion, increased hip internal rotation at initial contact, and reduced knee flexion coupled with increased knee internal rotation at the peak of knee flexion [23,24,25,26,27].

Three-dimensional motion analysis is a comprehensive method for identifying landing risk factors; however, its extensive time requirements and higher costs limit its practicality. In contrast, the landing error scoring system (LESS) evaluates landing techniques using two-dimensional video images, making it clinically feasible and cost-effective. As a screening tool for ACL injury, the LESS demonstrates good sensitivity (86%) and specificity (64%) [18,28]. It identifies high-risk movement patterns and faulty landing biomechanics, such as increased hip internal rotation and adduction during initial contact, increased knee valgus, and reduced hip and knee flexion. An LESS score of 5 or more indicates a poor jump landing technique, correlating with a higher ACL injury risk ratio of 10.7 [28]. Previous studies have shown an increase in LESS scores for both sexes following functional exercise protocols, indicating a poorer landing technique [29]. Additionally, research by Van Melick et al. and Gokeler et al. revealed increased LESS scores after fatigue protocols, particularly in high-risk individuals such as those with an ACL reconstruction history [30,31].

Dynamic tape, developed by Ryan Kendrick, offers greater elasticity and resistance compared to rigid or Kinesio tape without restricting joint movement [32]. These characteristics allow for its use in daily activities or competitive games without restricting joint movement. Robinson et al.’s study shows its potential to reduce hip adduction moments and angles in patients with greater trochanteric pain syndrome [33]. However, clinical research on dynamic tape is extremely limited. Our previous research indicates that dynamic taping, especially with the spiral technique, reduces LESS scores in high-school volleyball athletes, particularly those at high risk [34]. Thus, dynamic taping holds promise for influencing lower-limb biomechanics. This study aims to investigate the effects of dynamic taping on landing biomechanics in fatigued athletes. We hypothesize that dynamic taping can mitigate adverse changes in lower-limb biomechanics following fatigue. Insights from this study may provide a novel approach to improving lower-limb biomechanics and reducing ACL injury rates. 

## 2. Materials and Methods

### 2.1. Participants

Twenty-seven high-school football athletes were recruited for this study between January and November 2022. The sample size for this study was calculated using an a priori power analysis conducted with the G*Power software (Version 3.1.9.6; Heinrich-Heine-Universität Düsseldorf, Düsseldorf, Germany) based on our previous research that demonstrated an effect size of 0.75 with dynamic taping [34]. Given this effect size, an alpha level of 0.05, and a desired power of 0.8, we determined that a minimum of 16 participants were required. The inclusion criteria for this study were as follows: (1) high-school football athletes competing in Taiwan’s Division One league; and (2) aged between 15 and 18 years. The exclusion criteria included the following: (1) participants who had received lower-limb surgery within the past year; (2) participants with acute medical conditions that would preclude participation in football training and competition; (3) participants who had sustained a traumatic brain injury within the past six months; (4) participants who had experienced vestibular impairments in the past six months; (5) participants with allergies to dynamic tape; and (6) pregnancy. The study’s protocol was approved by the Chang Gung Memorial Hospital Institutional Review Board (approval no. 202002434B0) and registered with the Clinical Trial Registry (https://clinicaltrials.gov/—U.S. National Library of Medicine #NCT05288296). All participants and their legal guardians provided written informed assent and consent. 

### 2.2. Dynamic Taping

**Figure 1:**
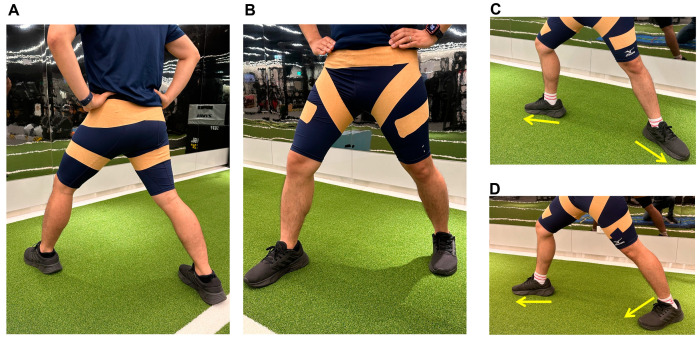
Demonstration of the dynamic tape applied to the bilateral hip. (**A**) Posterior view, (**B**) frontal view, (**C**,**D**) comparison of active and sham taping. The active and sham dynamic taping was applied directly to the skin over both hip joints. Starting from the anterior middle thigh, it wrapped around the thigh in an upper lateral direction to the posterior thigh, then continued to the proximal medial thigh, and subsequently wrapped below the anterior superior iliac crest in an upper lateral direction. After that, the tape crossed the lower back to the contralateral lower quarter of the abdomen. In active taping, the hip was positioned in 40° abduction, 20° extension, and full external rotation (**C**). In contrast, the hip was positioned without abduction and external rotation during sham taping (**D**).

The active and sham dynamic taping methods were used by the same experienced athletic trainer and applied directly to the skin (Figure 1). In the active taping method, a spiral double layer of 7.5 cm (Powerband) Dynamic tape^®^ (Posture Pals Pty LTD, Hangzhou, China) was applied to the bilateral hips with the hips positioned in a 40° abduction, 20° extension, and full external rotation. This was aimed at resisting hip adduction, flexion, and internal rotation. Sham taping, on the other hand, was applied without inducing hip abduction and external rotation. The dynamic tape was applied bilaterally to evaluate LESS items such as stance width, lateral trunk flexion, and overall impression. The powerband was created by applying additional lengths of dynamic tape in parallel [32]. 

### 2.3. Landing Error Scoring System

The LESS is a field screening field test designed to evaluate individual landing techniques through 17 items [28]. Before the task, all the participants were allowed as many practice trials as needed, but no feedback or coaching on their landing strategy was provided during the task. For the task, the participants jumped from a 30 cm high box to a designated landing area, a distance equivalent to 50% of their body height, and they immediately performed a vertical jump as high as possible. All the subjects completed three trials of the jump landing task before and after the fatigue protocol.

Two standard Handycams (DCR-CX900, Sony Group Corporation, Tokyo, Japan) captured the frontal and lateral views of the entire jump landing test. To rate the LESS scores, we reviewed the frontal and sagittal views frame by frame. These frames included the lower limb position, posture, and foot position at initial foot contact (Items 1–8), maximal knee flexion (Items 9 and 10), and the symmetry of landing (Item 11). Next, the trunk and lower-limb joint displacement from initial contact to maximal knee flexion were assessed (Items 12–15). Finally, the joint displacement and the overall impression of the entire landing task were evaluated (Items 16 and 17). Participants who scored an error in more than two of the three trials were marked with an error; otherwise, the individual item was coded as no error. The LESS scores were rated independently by an experienced rater, focusing primarily on the dominant leg. This rater, a physical medicine and rehabilitation physician, had over 10 years of experience in sports medicine and functional movement evaluation. The intra-rater reliability of this rater was high ((ICC)_2,1_ = 0.916). The rater was blinded to the randomized allocation of the active and sham taping.

### 2.4. Functional Agility Short-Term Fatigue Protocol

**Figure 2:**
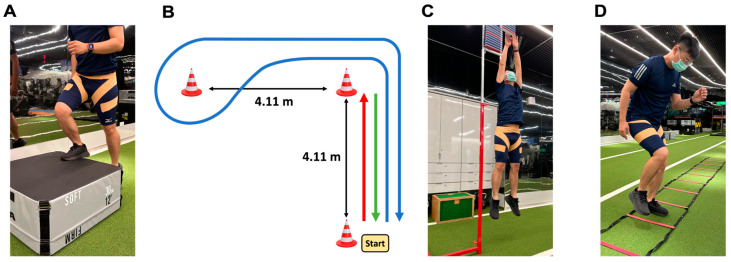
Functional agility short-term fatigue protocol. The participants performed four exercises consecutively without rest: (**A**) step-ups, (**B**) L-drill, (**C**) countermovement jump, and (**D**) agility ladder drill. They had to complete a total of four sets.

The functional agility short-term fatigue protocol (Figure 2) consists of the following series of agility drills selected for their relevance to common athletic skills in football [25]: (1) Step-ups: The participants performed step-ups on a 30 cm box for 20 s at a pace set by a metronome at 200 beats per minute. (2) L-drill: Three cones were arranged to form an ‘L’ shape with each cone 4.11 m apart. The participants sprinted to one cone, returned to the starting cone, and repeated the sprint before running around the second cone, cutting left to the third cone. They then circled the third cone, ran around the second cone, and sprinted back to the start. (3) Countermovement jump (CMJ): The participants performed five consecutive CMJs, aiming to reach 80% of their maximum jump height with each leap. (4) Agility ladder drill: Using a 6 m ladder, the participants performed the drill to the rhythm of a metronome set at 200 beats per minute. For the first and third run, they began at one end, facing the ladder, and quickly stepped into and out of the rungs with alternating feet, repeating this pattern until they reached the other end and then reversing direction. For the second and fourth run, the participants started perpendicular to the ladder and stepped laterally into and out of the rungs, again with alternating feet, until they reached the end and then returned. According to previous research, the participants had to complete all four sets of the protocol consecutively to reach fatigue status, with the entire sequence lasting approximately 6 min [25,27,35,36].

### 2.5. Research Design

**Figure 3:**
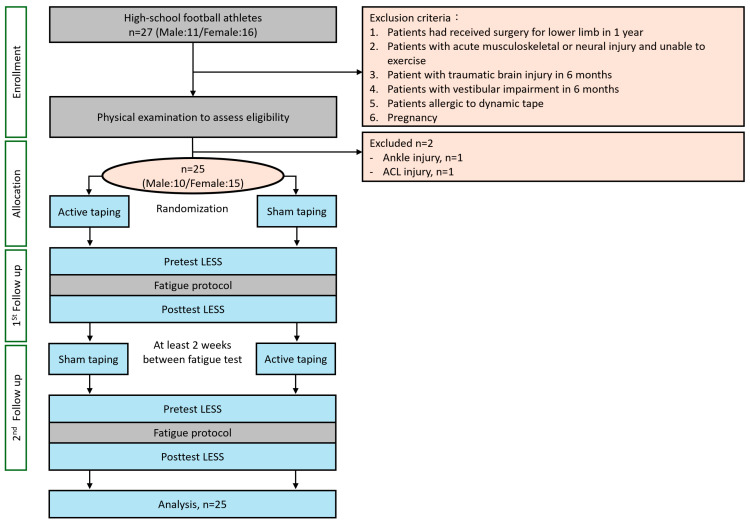
Experiment flowchart.

This was a single-blind, randomized controlled crossover trial adhering to the CON-SORT 2010 guidelines (Figure 3). After a comprehensive physical assessment, two participants were excluded due to ankle and knee injuries. Twenty-five participants were included in the study and randomly allocated via simple randomization by drawing lots to two sequences: (A) active taping followed by sham taping; and (B) sham taping followed by active taping. Opaque sealed envelopes were utilized to ensure allocation concealment. After taping, the participants underwent a fatigue protocol and were evaluated using the LESS before and after the protocol. Each taping session was separated by a two-week period to prevent interference from discomfort following the fatigue protocol and the effects of dynamic taping. Ultimately, all 25 participants completed the study without any loss to follow-up or discontinuation of the intervention (Table 1). The LESS scores were independently rated by an experienced rater. The sum and individual items of the LESS, along with subgroup analyses for high- and low-risk participants, were further analyzed.

### 2.6. Statistical Analyses

A Shapiro–Wilk test was conducted to assess the normality of the measurements in the LESS. The results confirmed that the LESS scores for both the active and sham taping groups were normally distributed. We employed the paired *t*-test to compare the effects of the fatigue protocol on the LESS total score and each specific scoring item both before and after the sham and active dynamic taping. We also used independent *t*-tests to compare the pre–post difference between the sham and active groups. All statistical awere performed using IBM SPSS (Version 24.0; IBM Corporation, Armonk, NY, USA). Cohen’s d effect sizes were calculated using G*Power to aid in interpreting the results. The magnitudes of the effect sizes were categorized using Cohen’s thresholds: 0.0 to 0.19 as trivial; 0.20 to 0.49 as small; 0.50 to 0.79 as moderate; and above 0.80 as large [37]. The level of significance for all statistical tests was set at 0.05.

## 3. Results

**Figure 4:**
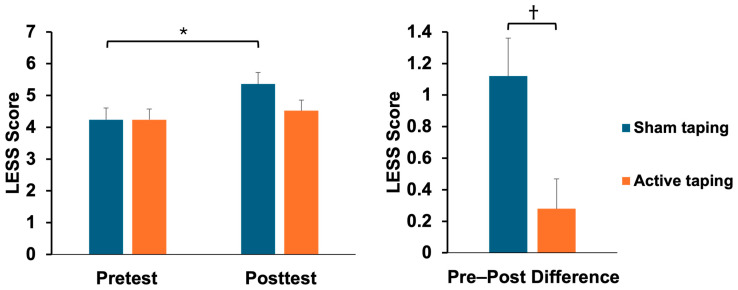
Comparison of LESS scores between sham and active dynamic taping following a fatigue protocol (mean ± SE). * = *p* < 0.05, † = *p* < 0.01.

After the fatigue protocol, the LESS scores of the athletes who received the sham taping increased from 4.24 ± 1.83 to 5.36 ± 2.00 (t = −2.07, *p* = 0.04, effect size = 0.61). In contrast, the active taping group did not show a significant statistical change following the fatigue protocol. Furthermore, the difference in the LESS scores between the sham and active taping groups following the fatigue protocol was statistically significant (sham taping: 1.12 ± 1.20; active taping: 0.28 ± 0.94, p = 0.007) (Table 2, Figure 4).

For the specific LESS items, the frequency of a faulty landing strategy in the sham taping group increased for trunk flexion displacement and joint displacement (Items 14 and 16) after the fatigue protocol. In contrast, the active taping group did not exhibit any statistically significant changes (Table 3). Additionally, the difference in the medial knee position at initial contact (Item 5) between the sham and active taping groups following the fatigue protocol reached statistical significance (sham taping: +0.16 ± 0.47; active taping: −0.12 ± 0.44, *p* = 0.04).

**Figure 5:**
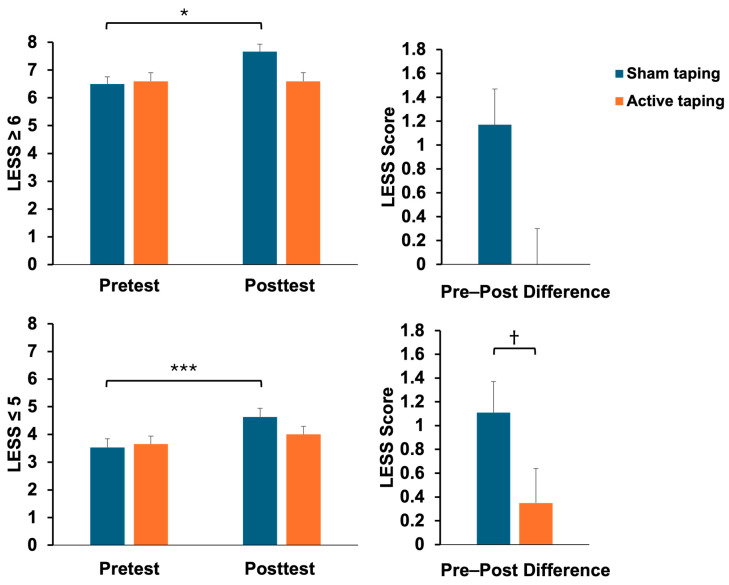
Comparison of LESS scores in high- and low-risk groups between sham and active dynamic taping following a fatigue protocol (mean ± SE). * = *p* < 0.05, *** = *p* < 0.001, † = *p* < 0.05.

In the high-risk group (LESS ≥ 6), the LESS scores for the athletes who received sham taping increased from 6.50 ± 0.84 to 7.67 ± 1.03 (*t* = −2.91, *p* = 0.03, effect size = 1.63). In the low-risk group (LESS ≤ 5), the LESS scores for the athletes who received sham taping also rose from 3.53 ± 1.43 to 4.63 ± 1.67 (*t* = −4.59, *p* < 0.001, effect size = 1.15). Conversely, the active taping group did not demonstrate a significant statistical change following the fatigue protocol. Furthermore, in the low-risk group, the pre–post difference between the sham and active taping groups following the fatigue protocol was statistically significant, with the sham taping group showing an increase of 1.11 ± 1.50 compared to 0.35 ± 1.27 in the active taping group (*p* = 0.03) (Table 4, Figure 5).

## 4. Discussion

The results indicated a significant increase in the LESS score following the fatigue protocol. Additionally, dynamic taping using the spiral technique appeared to mitigate faulty landing biomechanics in the fatigued athletes, such as decreased hip and knee flexion and increased hip internal rotation during landing (Table 3). The impact of the dynamic taping was demonstrated across the high- and low-risk groups (Table 4). To our knowledge, this is the first study to explore the effects of dynamic taping on landing biomechanics before and after a fatigue protocol. Therefore, dynamic taping may potentially offer protective benefits in landing mechanics to prevent ACL injuries.

In a previous study [25], decreased hip and knee flexion were noted at initial contact during landing via motion analysis, resulting in a stiffer and more extended posture. This faulty posture is a risk factor for ACL injury due to increased anterior tibial translation and shear force, which strain the ACL [35]. In our study, the fatigued athletes who received sham taping also exhibited a stiffer posture upon landing, as evidenced by the increased trunk flexion displacement (Item 14) and joint displacement (Item 16). In contrast, these changes in landing biomechanics were not observed in the fatigued athletes who received active taping.

Fidai et al. demonstrated that the knee valgus increased during the drop jump test in youth athletes after a short-term fatigue protocol [38]. This increase in the knee valgus was noted at initial contact and at maximal knee flexion in the fatigued athletes [25,35]. In the current study, the difference in the medial knee position at initial contact (Item 5) between the active and sham taping groups reached statistical significance, but not in the medial knee displacement (Item 15), which shows the knee posture at maximal knee flexion. However, observational studies have shown that ACL injuries occur approximately 40 milliseconds after initial contact [39]. Therefore, dynamic taping could reduce the knee valgus at initial contact, potentially offering a protective effect.

Previous research has indicated that the minimal clinically important difference (MCID) for the LESS is approximately 1.16 [40]. In our study, the pre–post difference in the LESS scores in the sham taping group following the fatigue protocol was 1.12, which is very close to the MCID. In contrast, the difference in the LESS scores in the active taping group did not reach a significant change. Gokeler et al. reported that the LESS scores in both a healthy control group and high-risk patients, such as those who had undergone ACL reconstruction, increased after a fatigue protocol (from 2.5 to 6.0 in the control group; from 6.5 to 7.0 in the ACL reconstruction group) [30]. Our research presents similar results, with the LESS scores increasing significantly and closely approaching the MCID in both the high- and low-risk groups. However, compared to the low-risk group, the difference in LESS scores following the fatigue protocol in the high-risk group did not reach statistical significance. The possible explanations include the following: (1) a higher initial LESS score allows for less room for an increase, possibly creating a ceiling effect; (2) the small sample size in the high-risk group (only five participants) makes it difficult to achieve statistical significance.

A recent study showed that approximately two-thirds of ACL injuries occur in the first half of gameplay [41,42], suggesting that acute fatigue, typically resulting from high-intensity anaerobic exercise, may play a significant role [43]. In our current study, the functional agility short-term fatigue protocol, consisting of a series of agility drills in football, closely mimicked real game situations. Exercise training programs, such as the FIFA 11+ program for soccer athletes, have been proposed to prevent ACL injuries [44]. However, a meta-analysis revealed that while such programs decrease lower-limb injuries, they do not significantly reduce ACL injury rates. Additionally, compliance with ACL injury prevention programs among coaches and athletes has been found to be poor [45]. 

Biomechanical and neuromuscular factors are modifiable risk factors for ACL injuries and could be addressed through interventions such as Kinesio taping. However, studies have shown conflicting results. For instance, it was demonstrated that Kinesio taping did not reduce the knee valgus or lateral trunk lean during double-leg landings and jumps [46]. Conversely, another study found that Kinesio taping decreased the knee valgus at the initial contact of a double-leg landing in healthy male participants [47]. In our study, dynamic taping not only reduced the knee valgus at initial contact but also increased the trunk and joint displacement, particularly noted in Items 14 and 16 of our results, demonstrating its utility in correcting faulty landing biomechanics and potentially providing a supportive approach for athletes. Compared to Kinesio tape, dynamic taping is a relatively new technique with superior properties, including higher elasticity (dynamic tape: 200%; Kinesio tape: about 140%), multidirectional extensibility, absence of rigid endpoints, and stronger resistance and recoil [32]. Its application can produce a ‘boomerang effect,’ where potential elastic energy accumulated during concentric contraction is utilized as kinetic energy during the eccentric phase [48], making it a favorable option for correcting faulty landing biomechanics.

The strengths of this randomized controlled trial include the design, in which the participants served as their own controls. A sham group was established to minimize the placebo effect, and the rater was blinded to the allocation of the participants. However, this study has several limitations. First, although a sham group was incorporated, the absence of a non-taping group means that the study could not assess the impacts of psychological factors and proprioception separately. Second, the reliability and validity of the findings may be limited by the use of only one rater; involving multiple raters could have enhanced these aspects. Third, the small sample size may have hindered the achievement of statistical significance. Fourth, the duration of the effects of dynamic taping remains unknown. We only observed the immediate effects after the fatigue protocol without considering the duration of these effects and environmental factors.

## 5. Conclusions

The application of dynamic tape to the hip joint improves landing biomechanics by particularly reducing the knee valgus in initial contact and decreasing hip and knee extension during landing. This beneficial effect was observed in both the high- and low-risk athletes. Therefore, in clinical practice, dynamic tape could serve as a passive and supportive tool, providing protective benefits in landing mechanics to help prevent ACL injuries.

## Figures and Tables

**Table 1:** Demographic data of participants.

	Male (*n* = 10)	Female (*n* = 15)	*p*-Value
Age (Year)	16.40 ± 0.51	16.40 ± 0.52	1
Body Height (cm)	173.70 ± 3.16	161.43 ± 4.40	<0.001 *
Body Weight (Kg)	59.30 ± 5.33	55.92 ± 4.66	0.11
BMI (Kg/cm^2^)	19.67 ± 1.93	21.52 ± 2.30	0.048 *
Dominant Leg (Right/Left)	9/1	15/0	

BMI = body mass index. * Significant difference (*p* < 0.05) between male and female athletes. All data are reported as mean ± SD.

**Table 2:** The LESS scores of athletes between sham and active dynamic taping following a fatigue protocol.

LESS	Sham Taping (*n* = 25)	*p*-Value	*t*-Value	Effect Size	Active Taping (*n* = 25)	*p*-Value	*t*-Value	Effect Size	Δ (Post–Pre) between Groups *p*-Value
Pretest	4.24 ± 1.83	0.04 *	−2.07	0.61	4.24 ± 1.69	0.15	−1.50	0.46	
Posttest	5.36 ± 2.00				4.52 ± 1.69				
Δ (Post–pre)	1.12 ± 1.20				0.28 ± 0.94				0.007 †

Δ: Difference between pre and posttest. * Significant difference (*p* < 0.05) between pretest and posttest. † Significant difference (*p* < 0.01) of Δ between sham and active taping group. All data are reported as mean ± SD.

**Table 3:** The difference in individual items between sham and active dynamic taping following fatigue protocol.

LESS	Sham Taping				Active Taping				Δ (Post–Pre) between Groups *p*-Value
	Pretest (*n* = 25)	Posttest (*n* = 25)	Δ (Post–Pre)	*p*-Value	Pretest (*n* = 25)	Posttest (*n* = 25)	Δ (Post–Pre)	*p*-Value	
#1	0.64 ± 0.49	0.56 ± 0.51	−0.08 ± 0.49	0.43	0.40 ± 0.50	0.40 ± 0.50	0.00 ± 0.29	1.00	0.48
#2	0.00 ± 0.00	0.00 ± 0.00	0.00 ± 0.00	N/A	0.00 ± 0.00	0.00 ± 0.00	0.00 ± 0.00	N/A	N/A
#3	0.00 ± 0.00	0.04 ± 0.20	0.04 ± 0.20	0.33	0.00 ± 0.00	0.00 ± 0.00	0.00 ± 0.00	N/A	0.32
#4	0.64 ± 0.49	0.68 ± 0.48	0.04 ± 0.35	0.57	0.64 ± 0.49	0.52 ± 0.51	−0.12 ± 0.44	0.18	0.16
#5	0.24 ± 0.44	0.40 ± 0.50	0.16 ± 0.47	0.10	0.24 ± 0.44	0.12 ± 0.33	−0.12 ± 0.44	0.18	0.04 †
#6	0.04 ± 0.20	0.00 ± 0.00	−0.04 ± 0.20	0.33	0.00 ± 0.00	0.00 ± 0.00	0.00 ± 0.00	N/A	0.32
#7	0.00 ± 0.00	0.04 ± 0.20	0.04 ± 0.20	0.33	0.04 ± 0.20	0.04 ± 0.20	0.00 ± 0.00	1.00	0.32
#8	0.48 ± 0.51	0.52 ± 0.51	0.04 ± 0.45	0.66	0.60 ± 0.50	0.52 ± 0.51	−0.08 ± 0.49	0.42	0.38
#9	0.00 ± 0.00	0.00 ± 0.00	0.00 ± 0.00	N/A	0.00 ± 0.00	0.00 ± 0.00	0.00 ± 0.00	N/A	N/A
#10	0.08 ± 0.28	0.12 ± 0.33	0.04 ± 0.35	0.57	0.12 ± 0.33	0.08 ± 0.28	−0.04 ± 0.20	0.32	0.33
#11	0.16 ± 0.37	0.28 ± 0.46	0.12 ± 0.60	0.33	0.32 ± 0.48	0.40 ± 0.50	0.08 ± 0.57	0.49	0.81
#12	0.00 ± 0.00	0.00 ± 0.00	0.00 ± 0.00	N/A	0.00 ± 0.00	0.00 ± 0.00	0.00 ± 0.00	N/A	N/A
#13	0.00 ± 0.00	0.00 ± 0.00	0.00 ± 0.00	N/A	0.00 ± 0.00	0.00 ± 0.00	0.00 ± 0.00	N/A	N/A
#14	0.28 ± 0.46	0.52 ± 0.51	0.24 ± 0.44	0.01 *	0.28 ± 0.46	0.40 ± 0.50	0.12 ± 0.33	0.08	0.28
#15	0.64 ± 0.49	0.80 ± 0.41	0.16 ± 0.47	0.10	0.56 ± 0.51	0.68 ± 0.48	0.12 ± 0.33	0.08	0.73
#16	0.32 ± 0.48	0.60 ± 0.50	0.28 ± 0.54	0.02 *	0.32 ± 0.48	0.52 ± 0.51	0.20 ± 0.50	0.06	0.59
#17	0.72 ± 0.46	0.80 ± 0.41	0.08 ± 0.40	0.33	0.72 ± 0.46	0.84 ± 0.37	0.12 ± 0.53	0.27	0.76

N/A = not applicable. Δ: Difference between pretest and posttest. * Significant difference (*p* < 0.05) between pretest and posttest. † Significant difference (*p* < 0.05) between sham and active taping group. Item 1: knee flexion: initial contact, Item 2: hip flexion: initial contact, Item 3: trunk flexion: initial contact, Item 4: ankle plantar flexion: initial contact, Item 5: medial knee position: initial contact, Item 6: lateral trunk flexion: initial contact, Item 7: stance width: wide, Item 8: stance width: narrow, Item 9: foot position: external rotation, Item 10: foot position: internal rotation, Item 11: symmetric initial foot contact: initial contact, Item 12: knee flexion displacement, Item 13: hip flexion displacement, Item 14: trunk flexion displacement, Item 15: medial knee displacement, Item 16: joint displacement, Item 17: overall impression. All data are reported as mean ± SD.

**Table 4:** Subgroup analysis of the high- and low-risk groups after sham and active taping.

LESS	Sham Taping (*n* = 25)	*p*-Value	*t*-Value	Effect Size	Active Taping (*n* = 25)	*p*-Value	*t*-Value	Effect Size	Δ (Post–Pre) between Groups *p*-Value
LESS ≥ 6									
Pretest	6.50 ± 0.84	0.03 *	−2.91	1.63	6.60 ± 0.86	1.00	0	N/A	
Posttest	7.67 ± 1.03				6.60 ± 1.14				
Δ (Post–pre)	1.17 ± 0.98				0.00 ± 1.00				0.08
LESS ≤ 5									
Pretest	3.53 ± 1.43	<0.001 ***	−4.59	1.15	3.65 ± 1.27	0.17	−1.73	0.51	
Posttest	4.63 ± 1.64				4.00 ± 1.38				
Δ (Post–pre)	1.11 ± 1.50				0.35 ± 1.27				0.03 †

Δ: Difference between pretest and posttest. * Significant difference (* = *p* < 0.05, *** = *p* < 0.001) between pretest and posttest. † Significant difference (*p* < 0.05) between pre–post difference of sham and active taping. All data are reported as mean ± SD.

## Data Availability

The data supporting the conclusion of this study are available from the corresponding author on reasonable request.
